# Mass screening of rice mutant populations at low CO_2_ for identification of lowered photorespiration and respiration rates

**DOI:** 10.3389/fpls.2023.1125770

**Published:** 2023-03-03

**Authors:** A.N.M. Mubarak, A.J. Burgess, K. Pyke, W.P. Quick, E.H. Murchie

**Affiliations:** ^1^ Division of Plant and Crop Sciences, School of Biosciences, University of Nottingham, Leicestershire, United Kingdom; ^2^ Department of Biosystems Technology, Faculty of Technology, South Eastern University of Sri Lanka, University Park, Oluvil, Sri Lanka; ^3^ International Rice Research Institute, Los Baños, Laguna, Philippines

**Keywords:** rice, photosynthesis, photorespiration, mutants, gamma, EMS, phenomicsIntroduction

## Abstract

**Introduction:**

Identifying rice (*Oryza sativa*) germplasm with improved efficiency of primary metabolism is of utmost importance in order to increase yields. One such approach can be attained through screening genetically diverse populations under altered environmental conditions. Growth or treatment under low carbon dioxide (CO_2_) concentrations can be used as a means of revealing altered leaf photorespiration, respiration and other metabolic variants.

**Methods:**

We developed a pipeline for very high throughput treatment of gamma- and ethyl methanesulfonate- (EMS) induced mutant populations of IR64 rice seedlings at very low CO_2_ for 7 days. 1050 seedlings per batch at 5^th^ leaf stage were exposed to 60 ppm CO_2_ for the first day and 30 ppm for the remaining three days. Following this, putative candidates were identified by measuring chlorophyll depletion using SPAD. Screening results showed a distinct difference between the mutants and the WTs.

**Results and discussion:**

The mean chlorophyll loss in WTs ranged from 65% to 11% respectively, whereas in the mutant lines chlorophyll loss ranged from 0 to 100%, suggesting considerable phenotypic variation. Rice mutants with a reduced chlorophyll reduction (<10%) were identified as ‘Chlorophyll retention mutants’ (CRMs) under low CO_2_ stress. In total, 1909 mutant lines (14,000 seedlings) were screened for chlorophyll content under 30 ppm CO_2,_ with 26 lines selected for detailed screening. These 26 putative candidates were self-seeded to produce an M_5_ generation, used to determine the genetic control of the altered response to low CO_2._ Gas exchange of light and CO_2_ response revealed that there were significant variations among photosynthetic properties in two selected rice mutants. The CO_2_ compensation points in the absence of photorespiration and leaf respiration rates were lower than the WTs and anatomical analyses showed that CRM 29 had improved mesophyll cell area. We propose that this approach is useful for generating new material for breeding rice with improved primary metabolism.

## Introduction

The global population is projected to rise to between 9.7 and 11 billion by 2050 (World Population Prospects, 2022). Currently, approximately 50% of the world’s population grow and consume rice (*Oryza* sp.) as a staple crop. Although the annual rice production is above 750 million tonnes (FAO, 2020), it is expected the rice yield potential has to be increased by 30% over the next 30 years to avoid food shortage (Baldwin et al., 2012; World Population Prospects, 2022).This is further confounded by the predicted climatic changes and competition for land for urbanisation. For many cereal crops, the gains of first Green Revolution are largely exhausted (Long et al., 2006) and the Harvest Index has almost reached its maximum level (0.56) in most of elite rice cultivars (Cassman, 1999; Yang and Zhang, 2010; Furbank et al., 2020). Therefore, finding alternative avenues to improve yield production is a key target for food security.

Photosynthesis is a primary determinant of crop yield: the efficiency by which a crop captures light and converts it into biomass over the growing season therefore represents key targets for yield improvement (Long et al., 2006). However, some of the processes involved in primary metabolism have not traditionally been a direct target for breeding programmes (Sharwood, 2017). A number of recent studies have provided strong evidence that improving the photosynthetic processes through genetic engineering can lead to increases in biomass and yield. This can be achieved through the overexpression of a single enzyme, including those involved in the Calvin Benson cycle such as Sedoheptulose-1,7-bisphosphatase (SBPase: EC 3.1.3.37)(Ding et al., 2016; Driever et al., 2017; Simkin et al., 2017a) and transketolase (TK; EC 2.2.1.1) (Khozaei et al., 2015; Suzuki et al., 2017); in electron transport (Simkin et al., 2017b; Yadav et al., 2018); or in photorespiration (López-Calcagno et al., 2019). Alternatively, genes incorporated from other species such as cyanobacteria (Tamoi et al., 2006; Uematsu et al., 2012; Köhler et al., 2017) or wild relatives (Placido et al., 2013; Prins et al., 2016; McAusland et al., 2020) have been shown to increase biomass. Finally and importantly, variation may also exist within existing germplasm (Sharwood et al., 2022; Cowling et al., 2022).

The establishment of natural genetic variation in these complex traits is essential for the provision of markers and lines to be used in breeding programmes. As opposed to targeting known genes or pathway, genetic variation may be induced from stable lines generated from mutagenised seed. Rice mutant populations have been used in the past to generate useful variation in disease and pesticide resistance (Steuernagel et al., 2016; de Andrade et al., 2018) as well as resistance to a number of abiotic stressors (Nakhoda et al., 2012; Joshi et al., 2016; Zeng et al., 2018; Hwang et al., 2020). The International Rice Research Institute (IRRI), Manilla, the Philippines developed collections of seeds mutagenised by Gamma ray (GR) at 250 GY, and by fast neutron (FN) at 33 GY irradiance levels (Wu et al., 2005). Lines subject to chemical mutagenesis with Diexpoxybutane (DEB) and Ethylmethanesuphonate (EMS) are also in existence. The GR and FN treatment creates point mutations in the rice genome, whereas the DEB and EMS treatment cause larger deletions. It is assumed the point mutation might create forty-point mutational hits in each mutant plant (Krishnan et al., 2009) which is believed to be a reasonable number for subsequent genetic analysis. Furthermore, the analysis of point mutations are relatively easier than the larger deletion mutants. More than 38,000 mutants were advanced to M_4_ generation (Wu et al., 2005). A subset of these mutant collections (12500 lines) have been successfully screened for increased vein densities and corresponding reduced leaf width with five lines identified and used as genetic stock for the global C4 Rice Consortium (IRRI, 2006; Feldman et al., 2014). In addition to increased vein density, the promising mutants exhibited improved photosynthetic characteristics including higher light saturated photosynthetic capacity per unit leaf area, higher maximum carboxylation rates, dark respiration rates and electron transport capacities (Feldman et al., 2017). Moreover, another subset of GR and DEB mutants have been screened for leaf anatomical features and revealed that leaf interveinal distance depends on mesophyll cell size rather than the numbers within the leaf veins (Smillie et al., 2012).

Whilst generating variation in photosynthetic traits has been achieved, high throughput screening can be difficult. Gas exchange is frequently used for measurement of photosynthesis and respiration at high resolution but is slow and not feasible for thousands of lines with replication (Cowling et al., 2022). One solution is to utilise large screening chambers which enable a large number of plants to be exposed to modified environmental conditions simultaneously. For screening for photosynthetic variation, reducing the CO_2_ concentration in the growth chamber is able to generate a condition close to the CO_2_ compensation point and hence enable survival selection. This can be used to identify more efficient photosynthetic pathways or enzymes. Leaves which possess lowered rates of photorespiration could be identified in this way. For example, C4 species generally have a lower CO_2_ compensation point compared to C3 species, due to the existence of a CO_2_ concentrating mechanism that eliminates photorespiration. Therefore, C4 species will generally perform better than C3 species under low CO_2._ This approach was successfully applied to a screen of mutants of the C4 plant *Setaria viridis* where a loss of function of PSII identified by chlorophyll fluorescence identified increased CO_2_ compensation points (Coe et al., 2018) and later one gene candidate as carbonic anhydrase (Chatterjee et al., 2021). Historically, low CO_2_ has been applied in historic screening experiments using a variety of plants and crops including *Arabidopsis thaliana* (Badger et al., 2009), soybean (*Glycine max*) (Widholm and Ogren, 1969), wheat (*Triticum aestivum*) and soybean (Menz et al., 1969), tall fescue forage grass (*Festuca arundinacea*) (Nelson et al., 1975) and tobacco (*Nicotiana* sp.) (Medrano et al., 1995). However, there are other ways in which low CO_2_ treatment can be usefully applied within screens for plant metabolism. Under low CO_2_ the restricted activity of the Calvin Benson cycle increases excitation ‘pressure’ on photosystems and on the thylakoid electron transport chain leading to multiple stress processes. Depending on treatment conditions, these may include photooxidative stress and the generation of reactive oxygen species but also excessive carbohydrate consumption. In the latter, substantially reduced or net zero photosynthetic rates will result in tissue death.

Genetically diverse populations require a means of assaying for key physiological traits in very large numbers of plants simultaneously. With biological replication this can mean several thousand plants or more. With this in mind, we developed and applied a pipeline using chambers at a very low CO_2_ levels (30 – 60 ppm) to repetitively screen a large mutant rice population over relatively short periods of 7 days analyse new traits for improved primary metabolism. Our approach was to identify properties that lead to greater resilience under such conditions, but which may have their origins in primary metabolic properties such as respiration, photosynthesis, photorespiration and oxidative stress tolerance. To provide a tractable and broad assay we used chlorophyll loss as a marker for such resilience. In addition to the mutant and parental WT rice, the C3/C4 intermediate *Panicum miliodes* and the C4 type *Echinochloa glabrescens* provided a reference for more efficient photosynthesis under reduced CO_2_. We show that it is possible to reveal individual mutant phenotypes with altered leaf respiration, photorespiration and leaf architecture.

## Materials and methods

### High-throughput mass plant screening chambers

Plant screening trials were carried out at the International Rice Research Institute (IRRI), Los Baños, the Philippines. A sub set of 1,900 M_4_ GR-treated lines plus 672 M_4_ EMS mutant lines were selected from the larger collection of IR64 mutants (Wu et al., 2005), compared against the parental wild type (IR64-21), *Echinochloa glabrescens* (C_4_ type) and *Panimcum milioides* (C_3_-C_4_ intermediate type). Rice seeds were sown at the spacing of 2.5cm in plastic trays (24 x16cm x10 cm^3^) in soil media with fertilizer ratio of 1:1:1of N:P:K. Prior to this, the soil was sterilised at 80^0^ C for three days. Aside from pests and disease, this step minimises the effect of soil and animal respiration on photosynthetic screening. Each tray contained eight rows of 8 plants (8x8). Prior to screening, a ‘survey’ of glasshouse grown C3 and C4 responses to CO_2_ took place in order to validate the conditions that would be used (**Supplementary File 1**).

### Chamber optimisation and initial screening

Seedlings were grown and maintained in greenhouse facilities for two weeks. Foliar nutrients were applied before the rice seedlings were moved to the chambers. The chamber description is in **Supplementary data File 1** following Lape et al. (2008). WT seedlings (IR 64-21; *P milioides* and *E. glabrescens*) were used to optimise conditions within the chambers prior to mutant screening experiments. At 5^th^ leaf stage, 19 trays were introduced for each chamber run, which contained on average 1064 x parental (IR-64) rice seedlings 10-15 x *Panicum milioides* seedlings and 10 x *E. glabrescens* seedlings.

**Figure 1:**
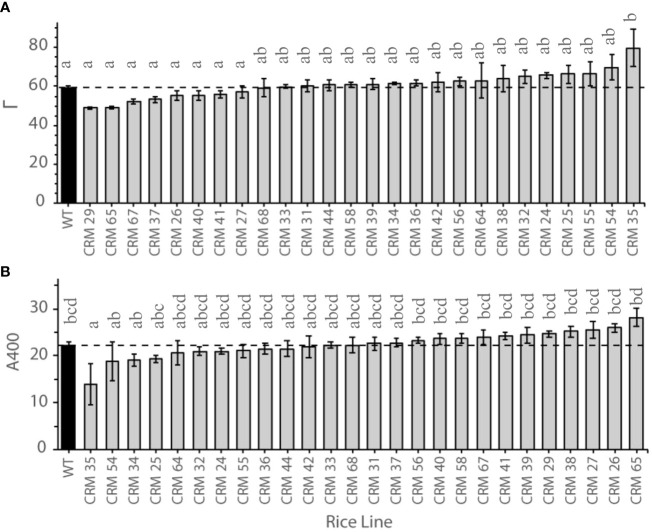
Photosynthetic traits of select Chlorophyll Retention Mutants (CRMs; grey) versus the parental wild type (WT; black) IR64, where the dotted lines indicates the WT level. (A) Mean CO2 compensation points (G) of 8th leaf in CRM mutant lines. Photosynthetic measurements were made on at least 3 plants per line. Twelve WT plant responses were included. Values are means and error bars represent standard error of the means. n.3. (B) Photosynthetic rate at ambient CO2 (A400) of 8th leaf in CRM mutant lines. Photosynthetic measurements were made on at least 3 plants per line. Twelve WT plant responses were included. Values are means and error bars represent standard error of the means. n.3. Letters indicate differences according to *post-hoc* Bonferroni test.

On day 1 in the chamber, CO_2_ concentrations were maintained at 60ppm followed by three days at 30ppm (**Figure 1**). Plant chlorophyll was quantified using a SPAD 502 meter (Minolta, Osaka, Japan), which was calibrated prior to measurements according to the manufacturer instructions. Total leaf chlorophyll content was measured by obtaining of an average of 3-5 SPAD readings per leaf, at two different time points: on the day before transfer to chamber and after three days of exposure to 30ppm CO_2_. Chlorophyll reduction (in %; Chl_red_) over the four-days of treatment were calculated using the following equation: (before/(before – after)) x 100.

Following this, mutants were screened along with WTs. Each soil tray contained evenly spaced 7 rows of mutants (8 plants per line) and one row of WTs. Fifteen rounds of trials during a four-month period were conducted with a total of 1064 rice seedlings from 133 mutant lines analysed each round. Accordingly, 133 mutant lines (1064 mutant seedlings) per chamber were run every four days. As such a total of 1909 mutant lines (approximately 13,500 seedlings) belonging to 1237 GR mutant lines (8700 seedlings) and 672 EMS mutant lines (4800 seedlings) were analysed.

Following this screen, putative mutant lines were selected based on lowest chlorophyll loss, where the mutants displaying <10% chlorophyll reduction selected and referred to as Chlorophyll Retention Mutants (CRMs). A total of 26 rice candidate mutant lines were identified (**Table 1**) and re-screened to-confirm the phenotype. These 26 x CRMs were removed from the chambers after four-days of screening treatment and transferred into plastic pots (10cm x 10cm x 10cm) which were filled with sterilized puddle soil. These were grown in the glasshouse with sufficient irrigation to allow them to self-seed and generate an M_5_ population for detailed photosynthetic and biomass trials.

**Table 1:** Chlorophyll content (in SPAD units) on the 5^th^ leaf of wild type (WT) IR64 plants, the C3/C4 intermediate *Panicum milioides*, the C4 type *Echonochloa glabrescens* and the rice mutant lines.

Plant	Mean SPAD value		Chl_red_ %	No. of seedlings
	Before	After		
**IR64-21**	32.49 ± 0.05	11.02 ± 0.1*	65.92 ± 0.31	4350
***P. milioides***	37.38 ± 0.15	32.89 ± 0.22*	11.92 ± 0.51	1027
***E. glabrescens***	35.47 ± 0.58	36.83 ± 0.54	–	53
**Mutant lines**	33.0 ± 0.03	14.19 ± 0.06*	56.52 ± 0.18	13509

The measurements were taken on prior to treatment and following four days of reduced CO_2_ (see materials and methods). Means and standard errors shown as well as chlorophyll reduction percentage (Chl_red_ %) and number of seedlings measured. * indicates statistical significance at P<0.001 according to ANOVA in the before and after chlorophyll content per line.

### Analysis of photosynthetic traits

Detailed photosynthetic trait analysis experiments were carried in controlled plant walk-in growth room facilities at University of Nottingham, Sutton Bonington Campus, UK. Illumination was provided by 400 W metal halide lamps with a photoperiod of 12 hours, temperature of 28 °C/26 °C (day/night) and relative humidity of 57% ± 7% throughout. Photosynthetic photon flux density (PPFD) was 400 µmol photons m^-2^ s^-1^ at plant height.

The mutagenized M_5_ CRM rice seeds were washed 3 to 4 times in de-ionized water to remove the germination inhibitors and fungicide treatments applied prior to seed storage. Between 5 and 8 viable seeds were transferred into 5cm petridishes (Nunclon; Sigma-Aldrich Co, USA) with damp filter paper (Whatman Ltd, Kent, UK). De-ionised water was added to1/3 height of the petridish and the dishes were sealed with Parafilm insulation tape (Alpha laboratories, Eastleigh, UK). The petridishes were then transferred into germination rooms with the same day, night temperatures, photoperiod and relative humidity as the growth room but a reduced light intensity of 250 µmol photons m^-2^ s^-1^ for one week, or until the3^rd^ leaf emerged. Seedlings were then transferred into media for hydroponic growth with a full complement of nutrients in light proof 20 L tubs (Hubbart et al., 2012). Each experiment was a randomised block design with four plants per CRM mutant line (four replicates). Each hydroponic tank was considered a block with four mutant lines at least one IR64 plant (control). Hence, four such tanks were arranged fully randomised and blocked. With such setups, seven rounds of subsequent growth room experiments were performed (26 CRM lines). The growth room chamber conditions were PAR 400 µmol m^-2^ s^-1^, temperature day/night was 28°/25°C, RH 50-60%.

Leaf gas exchange measurements (spot measurements, net CO_2_ assimilation (A) versus internal CO_2_ (Ci)- Aci and light response curves- LRC) were taken with a LI-COR 6400XT infra-red gas-exchange analyser (LI-COR, Nebraska) on the 8^th^ youngest fully expanded leaf. The block temperature was maintained at 30°C using a flow rate of 500 ml min^−1^ and relative humidity between 50-60% and light was provided by a combination of in-built red and blue light emitting diodes (LEDs).

### Spot measurements

Spot measurements were performed on leaves to obtain stable levels of photosynthesis, stomatal conductance (gs), transpiration rates and instantaneous water use efficiencies. CO_2_ concentration was maintained at 400 ppm and light intensity at 1000 μmol m^−2^ s^−1^. Leaves were allowed to stabilise prior to measurement, which generally occurred within 2 – 3 minutes.

### A/Ci analysis

For the *ACi* curves, leaves were exposed to 1000 μmol m^−2^ s^−1^ throughout using a combination of inbuilt red and blue LEDs. They were placed in the chamber at 400 ppm CO_2_ for a maximum of 2 min and then CO_2_ altered in the following steps: to 250, 100, 75, 50, 25, 400, 400, 600, 800 and 1200 ppm. All plants were exposed to the same pattern except for the C4-type WTs, *P. milioides and E. glabrescens* which were exposed to an additional 0 ppm CO_2_ step at the bottom of the curve.

The CO_2_ compensation points (Г) were calculated as the x- intercept of a linear regression through the five lowest intercellular CO_2_ values on each *ACi* curve (Vogan et al., 2007). The initial slope was given as a measure of maximum carboxylation efficiency (CE) and respiration rate (Ro) estimated by interpolation of liner regression to the Y-axis. Curve fitting was performed to estimate *Jmax*, *VCmax* and *TPU* (Sharkey et al., 2007).

### Light response curves

Light response curves were on the same leaves which were used to measure *ACi* curves. Prior to this, sufficient recovery time was given (24 hours) to each rice plant. Illumination occurred over a series of 10 photosynthetically active radiation (PAR) values (low to high), 0, 50, 75, 100, 200, 400, 600, 800, 1000, 1500 µmol m^-2^s^-1^, with a maximum of 2 min at each light level The CO_2_ concentration was maintained at 400 ppm.

Curve fitting *via* a non-rectangular hyperbola was used to estimate light compensation points (LCP) as the x-intercept, the quantum yield efficiency (QE) from the initial linear regression slope, light saturation point (LSP) and maximum photosynthetic rate (A_max_) in accordance with (Burgess et al., 2017). In addition, dark respiration rates (Rd) were measured as the point where the LEDs were completely turned off (PAR= 0). We use the terms Ro and Rd to distinguish the two different methods.

### CO_2_ compensation point in the absence of day respiration (Г*)

Corrected CO_2_ compensation point in the absence of day respiration (Г*) and mitochondrial respiration rates (Rd*) were also measured as previously described by Brooks and Farquhar (1985) for candidate lines selected for contrasting responses (CRM 27, CRM 29, CRM 35). Here, the initial points the *ACi* curves (0 to 250 ppm CO_2_) were measured with PAR values of 100, 250, 500 and 800 µmol m^-2^ s^-1^. These lines were plotted on the same graph in order to find the converging points. The X-value is given as the Г* and the corresponding Y-value is given as the Rd*.

### Mesophyll cell characteristics

The flag leaves from candidate line CRM 29 and the WT (IR64) were harvested to analyse leaf anatomical features. Leaf mesophyll cells were isolated as previously described by Smillie et al. (2012) including treatment of the leaves in 4% paraformaldehyde overnight at 4°C, subsequent treatment in 0.1M sodium ethylenediaminetetraacetic acid (NaEDTA) followed by heating at 60°C for 6 to 8 hours. The mesophyll cells were isolated by means of a mechanical shear force applied by tapping with forceps, and the isolated cells were captured images with x160 magnification. Mesophyll cell plan (i.e. perimeter) was analysed using ImageJ software (Rasband, W.S., ImageJ, U. S. National Institutes of Health, Bethesda, Maryland, USA).

### Statistics

Results were analysed using GenStat statistical package (22^nd^ Edition, VSN international). The data was tested for the assumptions of normality. Analysis of variance (ANOVA) using an unbalanced design were performed for photosynthetic traits with a *post hoc* Bonferroni test. Finally, correlation analysis was made between photosynthetic traits.

## Results

### Percentage chlorophyll reduction following low CO_2_ treatment

**Figure 2:**
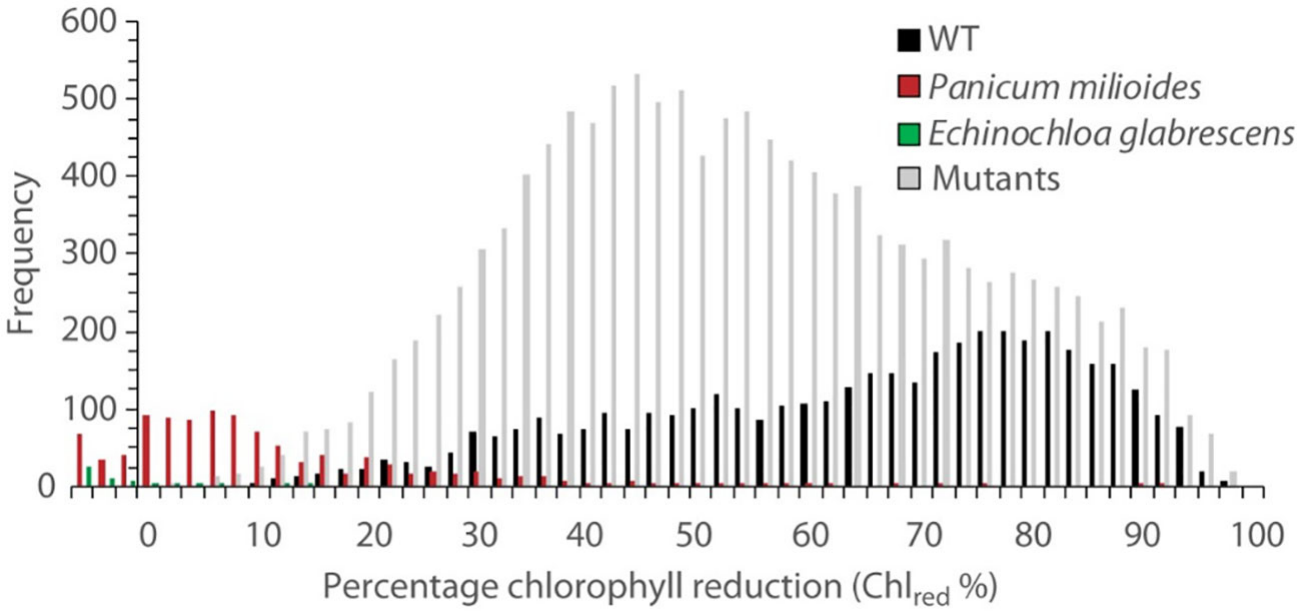
Frequency of chlorophyll reduction percentage (Chl_red_ %) in WT IR64 (black), the C3/C4 intermediate *Panicum milioides* (red), the C4 *Echinochloa glabrescens* (green) and the mutant rice population (grey). In total, 4261, 1018, 54 and 13,016 seedlings were screened before and after low CO2 treatment for WT, *P. milioides*, *E. glabrescens* and the mutants, respectively.

Transfer to 30 ppm CO_2_ arrested leaf growth and expansion for all WT rice lines in initial chamber optimisation experiments. According to SPAD measurements, the parental WT (IR64) seedings exhibited the largest reduction in chlorophyll content (Chl_red_) of 65.9% (P<0.001; **Table 1**; **Figure 2**). In comparison, the C_3_-C_4_ intermediate *P.milioides*, exhibited Chl_red_ of less than 10% (P<0.001) and the C4 type *E. glabrescens* exhibited a non-significant increase in chlorophyll content after treatment at low CO_2_. These preliminary experiments indicated that a threshold of 10% Chl_red_ provides a physiological meaningful cut-off point for identifying potentially interesting rice mutants.

In the mutant rice lines, Chl_red_ ranged from 8 to < 100% with an average loss of 53.1%, significantly different than the WT IR64 Chl_red_ (P<0.05; **Table 1**; **Figure 2**). Out of all lines screened, 26 mutant lines exhibited Chl_red_ of less than the 10% threshold, which encompasses 16 GR mutants and 10 EMS mutants (**Supplementary Table S1**). These promising mutant rice lines were called ‘Chlorophyll Retention Mutants’ (CRM) and represent 1% of the total rice seedlings screened. All further analyses were performed on the CRMs and WT IR64, only.

### Gas exchange parameters in CRMs

There were significant differences in photosynthetic traits between CRMs and the parental WT in response to altered CO_2_ concentrations (**Table 2**). The lines exhibited a range of CO_2_ compensation points (Γ), with 8 CRMs exhibiting a mean Γ of less than that of the WT (**Figure 1A**). Only one line, CRM 35, exhibited a Γ significantly greater than that of the WT (P<0.05). At ambient CO_2_ (400 ppm) there were no significant differences in assimilation rate (A400) apart from the same line, CRM 35, which exhibited a significantly lower A400 relative to WT (**Figure 1B**). Similarly, CRM 35 exhibited a significantly lower CE (**Table 2**). Stomatal conductance (gs) ranged from 0.38 mmol m^−2^ s^−1^ in CRM 35 to 0.95 mmol m^−2^ s^−1^ in CRM 29, with the latter exhibiting a significantly increased gs relative to WT (0.59 mmol m^−2^ s^−1^; **Table 2**). There were no significant differences in respiration (Ro) between the CRMs and the WT although values ranged from 4.73 µmol m^−2^ s^−1^ in CRM 64 to 8.57 in CRM 27 µmol m^−2^ s^−1^.

**Table 2:** Photosynthetic traits of chlorophyll retention mutant (CRM) rice lines relative to the parental wild type (WT) IR64 from spot measurements and ACi curves.

Genotype	A400**	Ci*	gs**	Г**	CE**	Ro**
WT	22.15 ± 0.78	314.60 ± 7.07	0.59 ± 0.03	58.76 ± 1.67	0.11 ± 0.01	6.45 ± 0.25
CRM 24	20.96 ± 0.72	326.07 ± 1.72	0.60 ± 0.03	65.58 ± 1.58	0.10 ± 0.01	6.35 ± 0.36
CRM 25	19.31 ± 0.77	322.39 ± 6.85	0.55 ± 0.06	66.22 ± 4.51	0.09 ± 0.01	6.20 ± 0.44
CRM 26	25.93 ± 0.94	337.30 ± 2.09	0.88 ± 0.05	55.19 ± 2.34	0.15 ± 0.01	8.41 ± 0.64
CRM 27	25.40 ± 1.79	305.55 ± 7.12	0.58 ± 0.05	57.25 ± 3.04	0.15 ± 0.01	8.57 ± 0.27
CRM 29	24.65 ± 0.62	339.90 ± 3.40	0.95 ± 0.05	49.02 ± 0.31	0.12 ± 0.01	5.75 ± 0.30
CRM 31	22.58 ± 1.49	313.06 ± 4.67	0.50 ± 0.03	60.32 ± 3.11	0.12 ± 0.01	7.54 ± 0.74
CRM 32	20.95 ± 0.83	320.43 ± 4.37	0.53 ± 0.03	64.97 ± 3.65	0.10 ± 0.00	6.26 ± 0.16
CRM 33	22.19 ± 0.60	319.88 ± 3.28	0.57 ± 0.03	59.87 ± 1.08	0.11 ± 0.01	6.53 ± 0.37
CRM 34	19.08 ± 1.37	313.78 ± 7.77	0.47 ± 0.08	61.68 ± 0.77	0.09 ± 0.01	5.55 ± 0.53
CRM 35	13.90 ± 4.30	291.96 ± 24.31	0.38 ± 0.07	79.83 ± 9.74	0.08 ± 0.02	5.56 ± 1.02
CRM 36	21.44 ± 1.21	322.50 ± 4.10	0.58 ± 0.06	61.75 ± 1.46	0.13 ± 0.01	6.78 ± 0.80
CRM 37	22.78 ± 1.02	324.87 ± 5.24	0.70 ± 0.04	53.27 ± 1.59	0.12 ± 0.01	6.24 ± 0.74
CRM 38	25.19 ± 1.13	307.48 ± 8.36	0.62 ± 0.04	63.99 ± 6.77	0.13 ± 0.01	8.40 ± 0.37
CRM 39	24.39 ± 1.66	323.10 ± 2.72	0.64 ± 0.03	61.14 ± 2.83	0.14 ± 0.01	8.50 ± 0.59
CRM 40	23.66 ± 1.17	318.78 ± 8.50	0.66 ± 0.13	55.24 ± 2.59	0.12 ± 0.01	6.49 ± 0.45
CRM 41	24.19 ± 0.84	312.17 ± 1.93	0.56 ± 0.03	55.90 ± 1.64	0.13 ± 0.01	7.21 ± 0.56
CRM 42	21.95 ± 2.25	296.23 ± 13.40	0.48 ± 0.12	62.32 ± 4.94	0.11 ± 0.01	6.78 ± 0.64
CRM 44	21.50 ± 1.76	305.91 ± 4.36	0.44 ± 0.04	60.75 ± 2.78	0.11 ± 0.01	6.70 ± 0.59
CRM 54	18.85 ± 4.10	319.09 ± 11.84	0.49 ± 0.14	69.87 ± 6.56	0.08 ± 0.03	5.35 ± 1.39
CRM 55	21.01 ± 1.36	337.88 ± 2.70	0.72 ± 0.03	66.71 ± 6.31	0.10 ± 0.00	6.67 ± 0.63
CRM 56	23.29 ± 0.56	326.14 ± 1.54	0.64 ± 0.03	62.50 ± 1.96	0.11 ± 0.00	7.01 ± 0.26
CRM 58	23.69 ± 0.99	325.01 ± 4.31	0.66 ± 0.05	60.95 ± 1.46	0.12 ± 0.01	7.29 ± 0.34
CRM 64	20.66 ± 2.63	309.11 ± 13.44	0.48 ± 0.11	63.06 ± 8.95	0.08 ± 0.03	4.73 ± 1.44
CRM 65	28.18 ± 1.89	331.61 ± 3.97	0.87 ± 0.04	49.19 ± 0.77	0.15 ± 0.01	7.40 ± 0.61
CRM 67	23.90 ± 1.61	324.61 ± 6.99	0.70 ± 0.04	52.20 ± 0.99	0.13 ± 0.00	6.71 ± 0.10
CRM 68	22.27 ± 1.57	314.15 ± 5.47	0.55 ± 0.07	59.17 ± 4.63	0.10 ± 0.01	5.82 ± 0.23
Mean CRM	22.38 ± 0.5	318.46 ± 2.32	0.61 ± 0.03	60.69 ± 1.31	0.11 ± 0.004	6.72 ± 0.20

Means and standard error of the means are shown where n≥3 for A400 (μmol m^−2^ s^−1^): Photosynthetic rate at 400 ppm CO_2_ concentration; Г (μmol mol^−1^): CO_2_ compensation point; CE (mol m^−2^ s^−1^): carboxylation efficiency; Ci (μmol mol^−1^): intercellular CO_2_ concentration; *gs* (mmol m^−2^ s^−1^): Stomatal conductance. * and ** indicates significant differences between lines according to ANOVA at P < 0.01 and P < 0.001, respectively (p<0.05). **Supplementary Table 2** shows letters indicating differences according to *post-hoc* Bonferroni test.

12 CRM lines were sub-selected for photosynthetic light response analysis. The maximum photosynthetic rates (A400) showed considerable variation, with CRM 35 exhibiting the lowest Amax at 21 µmol m^−2^ s^−1,^ significantly lower than that of the WT (P<0.05), and CRM 43 exhibiting the highest at 48.57 µmol m^−2^ s^−1^ (**Table 3**). In WT plants the mean light compensation point (LCP) was 42.68 µmol m^-2^ s^-1^. 8 of the mutant lines had an LCP below 40 µmol m^-1^ s^-1^, with 4 exhibiting values above that of WT, with the maximum being CRM 35 at 62.35 µmol m^−2^ s^−1^. Light saturation point (LSP) ranged from 600 µmol m^−2^ s^−1^ (CRM 38) to 1025.7 µmol m^−2^ s^−1^ (CRM 43). There was less variation in the quantum yield of photosynthesis (QE), with only CRM 35 exhibiting significantly lower QE relative to the WT. There were no significant differences in dark respiration (Rd) between all lines.

**Table 3:** Photosynthetic traits of chlorophyll retention mutant (CRM) rice lines relative to the parental wild type (WT) IR64-21 from light response curves (LRCs).

Genotype	A_max_**	QE**	LCP*	LSP*	Rd
**WT**	38.04 ± 1.04	0.06 ± 0.00	42.68 ± 2.71	688.17 ± 28.53	2.58 ± 0.21
**CRM 27**	36.63 ± 0.78	0.05 ± 0.00	34.50 ± 2.23	834.43 ± 43.53	1.63 ± 0.18
**CRM 29**	42.60 ± 0.15	0.06 ± 0.00	34.27 ± 4.94	702.00 ± 15.04	2.20 ± 0.35
**CRM 31**	39.90 ± 3.42	0.06 ± 0.01	34.33 ± 2.02	730.50 ± 110.63	2.08 ± 0.34
**CRM 32**	36.36 ± 2.83	0.06 ± 0.01	37.26 ± 7.23	654.40 ± 43.49	2.08 ± 0.22
**CRM 33**	34.86 ± 1.17	0.06 ± 0.00	32.98 ± 1.82	642.20 ± 29.36	1.88 ± 0.07
**CRM 34**	34.30 ± 2.27	0.06 ± 0.01	27.33 ± 3.99	689.50 ± 84.67	1.59 ± 0.39
**CRM 35**	21.00 ± 2.54	0.02 ± 0.00	62.35 ± 15.58	944.20 ± 19.67	1.67 ± 0.33
**CRM 36**	35.83 ± 2.85	0.06 ± 0.01	32.15 ± 2.07	681.00 ± 35.58	1.77 ± 0.13
**CRM 37**	32.90 ± 6.16	0.05 ± 0.01	49.20 ± 2.77	773.67 ± 98.00	2.34 ± 0.61
**CRM 40**	29.93 ± 1.88	0.04 ± 0.00	46.10 ± 9.04	828.75 ± 38.58	1.70 ± 0.24
**CRM 43**	48.57 ± 6.40	0.05 ± 0.01	31.47 ± 4.62	1025.67 ± 184.79	1.71 ± 0.58
**CRM 68**	31.10 ± 4.20	0.04 ± 0.01	52.53 ± 2.20	926.33 ± 144.18	2.03 ± 0.58
**Mean CRM**	35.33 ± 1.97	0.05 ± 0	39.55 ± 3.04	785.67 ± 36.38	1.89 ± 0.07

Means and standard error of the means are shown where n≥3 for A_max_ (μmol m^−2^ s^−1^): maximum assimilation rate; QE (mol mol^−1^): quantum use efficiency; LCP (μmol m^−2^ s^−1^): light compensation point; LSP (μmol m^−2^ s^−1^): light saturation point and; Rd (μmol m^−2^ s^−1^): dark respiration. * and ** indicates significant differences between lines according to ANOVA at P < 0.01 and P < 0.001, respectively (p<0.05). **Supplementary Table 3** shows letters indicating differences according to *post-hoc* Bonferroni test.

**Figure 3:**
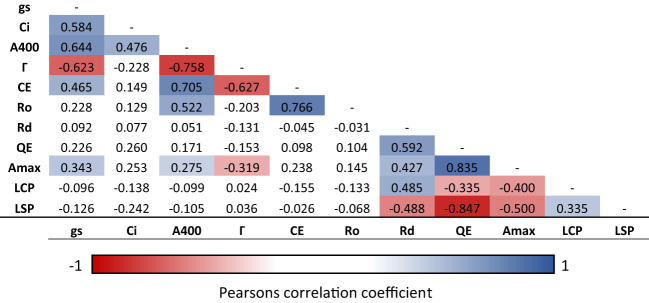
Correlation matrix of photosynthetic traits of rice WT and mutant lines. Traits include assimilation rate at ambient CO_2_ (A400), internal CO_2_ concentration (Ci) and stomatal conductance (gs) was calculated from spot measurements, CO_2_ compensation point (Γ), carboxylation efficiency (CE) and total respiration (Ro) calculated from ACi curves; and maximum assimilation rate (A_max_), quantum use efficiency (QE), light compensation point (LCP), light saturation point (LSP) and dark respiration (Rd) calculated for light response curves. Statistically significant (p < 0.05) negative linear correlations are shown in red, significant positive correlations are shown in blue, insignificant correlations are left blank.

Correlation analysis indicates a number of linked photosynthetic traits in response to a change in CO_2_ concentration and light intensity across the 12 selected CRMs and the WT (**Figure 3**). Г was significantly negatively correlated with A400 (r = 0.76, P<0.05), gs (r = 0.62, P<0.05), CE (r = 0.63,P<0.05) and Amax (r = 0.32, P<0.05). Although somewhat expected, this indicates that a reduced CO_2_ compensation point in rice leaves is associated with an increased ambient photosynthesis rates and carboxylation efficiency.

A significantly positive correlation was found between CE and A400 (r = 0.71, P<0.05) plus between QE and Amax (r = 0.84) suggesting improved quantum use- and carboxylation- efficiency increases ambient photosynthesis rates in rice leaves. However, a significant positive relationship was also found between CE and gs (r = 0.47, P<0.05) plus A400 and gs (r = 0.64, P<0.05) suggesting a potential trade-off with water loss.

### The origins of altered photosynthesis in candidate CRM rice lines

#### Photosynthetic features of CRMs

**Figure 4:**
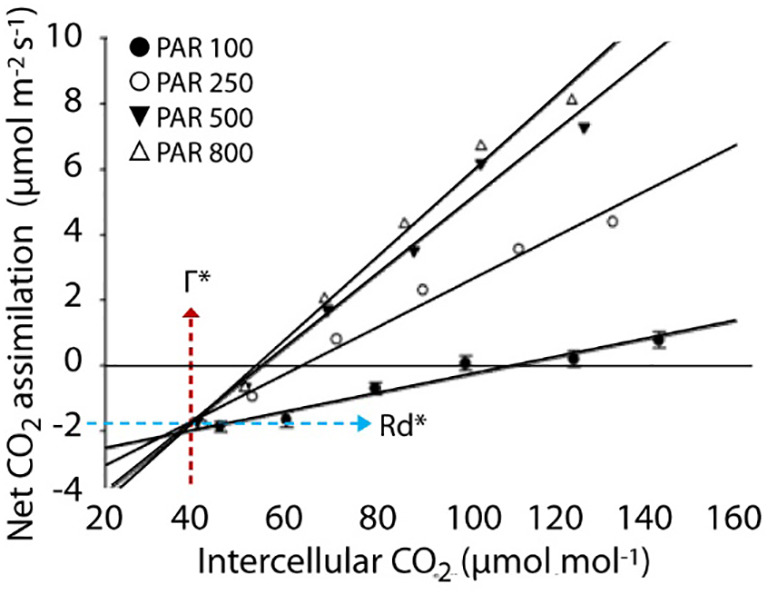
Measurement of corrected CO_2_ compensation point (Г*) and dark respiration in the light (Rd*) in rice plants following Brooks and Farquhar (1985).

Based upon the results obtained from ACi and LRCs in rice leaves, four mutant lines CRM 27, CRM 29, CRM 35 and CRM 65, were further investigated due to their variation in Γ and A400 relative to WT (**Figure 1**). One possible explanation for altered Γ is the influence of the rate of mitochondrial respiration in the light (Rd*) which can be calculated by the method of Brooks and Farquhar (1985) (**Figure 4**; **Table 4**). CRM 27 had a Г* of 40.44 µmol mol^-1^ which was not significantly different to WT plants at 42 µmol mol^-1^. In comparison, CRM 35 had a significantly higher Г* of 55.56 µmol mol^-1^ (P<0.001). There were no significant differences in Rd* between WT and CRM 35, but a significantly lower Rd* was seen in CRM 27.

**Table 4:** Corrected CO_2_ compensation point and dark respiration in the parental wild type (WT) IR64 compared to select Chlorophyll Retention Mutants (CRMs).

Genotype	Γ	Γ*	Δ_Γ-Γ*_	Rd	Rd*	Δ_Rd-Rd*_	n
**WT**	56.05 ± 0.75	42.04 ± 1.0	14.01	2.58 ± 0.21	1.84 ± 0.21	0.74	8
**CRM 27**	47.78 ± 1.25	40.44 ± 0.5	7.34	1.63 ± 0.18	1.04 ± 0.18	0.59	6
**CRM 29**	49.51 ± 1.5	41.78 ± 0.4	7.73	2.20 ± 0.35	1.59 ± 0.61	0.61	4
**CRM 35**	84.74 ± 5.59	55.56 ± 3.62	29.18	1.67 ± 0.33	1.81 ± 0.21	-0.14	5

Mean ± SEM for CO_2_ compensation point (Г), corrected CO_2_ compensation point in the absence of day respiration (Г*), difference in Γ and Γ* (Δ_Γ-Γ*_), dark respiration (Rd) dark respiration in the absence of mitochondrial respiration (Rd*), difference in Rd and Rd* (Δ_Rd-Rd*_) and *n* denotes number of plants measured. Significant differences were seen for all measured traits at P<0.01.

The discrepancy between Г and Г* showed interesting features. In CRM 27, a difference of 7.34 µmol mol^-1^ (47.2 – 40.44 µmol mol^-1^) was seen, CRM 35 showed a difference of 29.18 µmol mol^-1^ and for WT it was 14.0 µmol mol^-1^. This suggests mild changes in the mitochondrial respiration had great impact on determining CO_2_ compensation point in rice leaves. Whilst CRM 27 had reduced changes in Г* relative to WT, the mutant exhibited significantly lower Rd*. This suggests that CRM 27 might have changes in both mitochondrial respiration and the specificity of Rubisco for CO_2_/O_2_ as a result of the EMS mutation. In contrast CRM-35 had significant changes in the Г* and no significant changes in the Rd* suggesting this mutant may also contain altered properties of Rubisco or another unknown factor caused by the gamma irradiance treatment.

#### Anatomical features of CRMs

**Figure 5:**
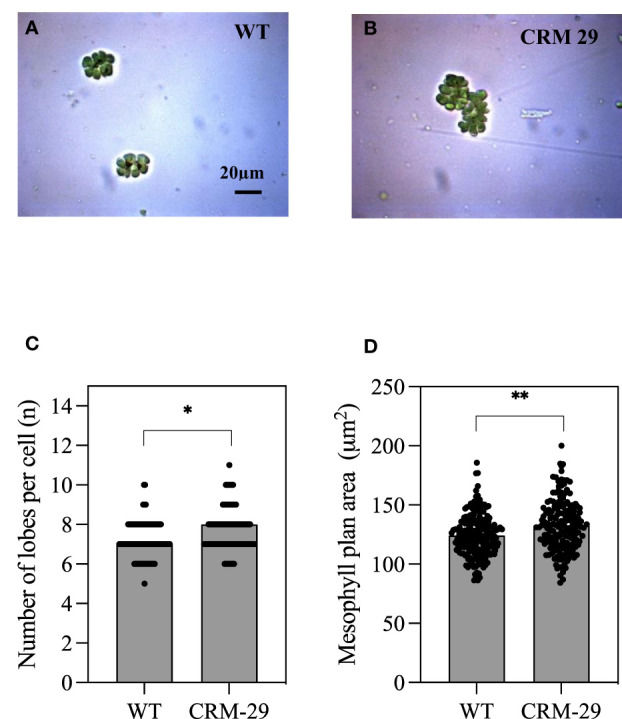
Isolated Mesophyll cells and associated traits of WT IR64 rice plants versus Chlorophyll Retention Mutant 29 (CRM 29). Isolated mesophyll cells from the flag leaves of **(A)** WT and **(B)** CRM 29. **(C)** Average number of lobes per mesophyll cell and **(D)** mesophyll plan area. Analyses were made on at least 170 isolated & cleared mesophyll cells. Values are means and error bars represent standard error of the means, n≥170. Mean ± SEM where * and ** indicate a significant at P<0.05 and P<0.01 according to an unpaired T-test, respectively.

Anatomical analysis was performed on WT and CRM 29, with example isolated mesophyll cells (MCs) shown in **Figure 5**. There were significant increases in the number of MC lobes in CRM 29 (7.7) relative to WT (7.2; **Figure 5C**; P<0.05). Further to this, the plan area (perimeter) of mesophyll cells (MCs) was significantly increased in plants of CRM 29 relative to WT (**Figure 5D**; P<0.01). An increase in MC plan area in flag leaves is beneficial in terms of accomodating a larger number of chloroplasts. This in turn may increase the number of active sites of Rubisco resulting in increased photosynthetic rates. Extensive lobing of MCs may also be useful in re-scavenging the photorespired CO_2_ in the leaves (Sage and Sage, 2009), thus may have contributed to the reduced *Vcmax* and Γ in CRM 29.

## Discussion

High throughput methods are required for the identification of improved yield and photosynthetic traits (Lawson et al., 2012; Murchie et al., 2018). Here we present the use of low CO_2_ chambers combined with rapid screening of pigment content to assess a large number of rice mutants for improved carbon metabolic efficiency. A comparable approach with similar plant numbers was successfully used to identify high CO_2_ compensation points in a mutant population of C4 species *S.viridis* and hence ‘reverts’ towards the C3 condition (Coe et al., 2018). Here we approach from the ‘other direction’ using chlorophyll content rather than fluorescence because most of C3 rice plants showed rapid chlorosis on exposure to low CO_2_ values and was the more sensitive assay. We seek a broader criterion than compensation point alone. We provide evidence that useful variation in photosynthetic and respiratory properties can be rapidly identified and that this provides a number of advantages over other methods, mainly as a result of the time constraints associated with analysing individual leaf level CO_2_ compensation points using an infra-red gas analyser. In contrast, a single large chamber experiment enables the screening of approximately 1060 seedlings per week, with a total of 14,000 rice seedlings across 15 consecutive runs.

### Species-dependent response to low CO_2_: Impact of Γ

Growth under low CO_2_ enables the identification of mutants with improved photosynthetic efficiency, which may be comparable to that of intermediate C3-C4 type plants such as *Panicum milioides*, used within this experiment. Survival in such an atmosphere generally requires a low CO_2_ compensation point to enable a positive carbon balance and indicates a potentially high maximum net photosynthetic rate (Downton and Tregunna, 1968; Menz et al., 1969). Measurements within *P. milioides* provided a cut off level of 10% chlorophyll reduction (Chl_red_) following 3 days at reduced CO_2_ as an indicator of potentially higher performing lines. Using this threshold, 26 mutant lines were identified (**Supplementary Table S1**) and termed chlorophyll retention mutants. This is a significantly reduced level compared to that of WT plants, which exhibit ~ 66% Chl_red_. It was hypothesised that post –treatment chlorophyll retention traits in rice CRM leaves were due to the alteration in chloroplast levels or mesophyll cell characteristics. Out of the 26 selected CRM lines, eight of the genotypes showed a non- significantly lower Γin their 8^th^ leaves than relative to the WT plants (**Figure 1**). This generally translated into a higher A400 and carboxylation efficiency (**Table 2**). This indicates that using SPAD as an initial screening step is effective in identifying lines with moderate changes in their photosynthetic properties.

The results presented here resemble the findings of Medrano and Primo-Millo (1985) performed on tobacco (*Nicotiana tabacum*) seedlings. They found that the tobacco plants that survived under low CO_2_ environments possessed significantly increased photosynthetic rates without corresponding changes in Γ. Thus, a reduction in Γ is not necessary for improved photosynthetic productivity, at least in tobacco and potentially for rice also. However, contrary to this, Cannell et al. (1969) found that survival of soybean (*Glycine max*) under low CO_2_ is dependent on a reduction in Γ. Furthermore, Cannell et al. (1969) suggested that the lack of variation in Γ for *Glycine* sp. corresponds with a lack of variation in net photosynthetic rates, indicating the link between the two traits. Similarly, Nelson et al. (1975) found that survival of tall fescue grass (*Festuca arundinacea*) under low CO_2_ is associated with a reduction in Γ.

### The potential of mutation- inducing treatments to alter photosynthesis in rice leaves

Rice possesses the smallest genome amongst the cereal crops, with an estimated size ranging from 400 Mb to 430Mb (Eckardt, 2000). Thus, chemical or radiation-based mutation provides a promising approach to identify beneficial gene targets. Of the identified candidate lines, CRM 27 and CRM 29 derive from EMS- induced mutation whereas CRM 65 was GY derived; the latter of which is expected to include large deletions or point mutations in the rice genome (Wu et al., 2005). The EMS- induced mutation is predicted to have targeted genes relating to chloroplast, mitochondria and peroxisome activity, as the study with CRM 27 showed significantly reduced Rd*. Although it is not clear which genes could influence both Г and Rd*, it is feasible that changes in PEPC could have occurred to create a type more similar to that seen in C4 species (Svensson et al., 2003). Further work is required to identify candidate genomic regions.

Alternative gene targets may have been those controlling cellular anatomy, particularly the mesophyll cell (MC) traits in flag leaves. It is possible this was one of the targets of mutation in CRM 29, resulting in significant increase in MC plan area and lobe number (**Figure 5**). Given the small differences shown here, we urge caution in attributing metabolic differences arising from this anatomical change. We also point out the differences in lobe number with Smillie et al. (2012) where different lines were used within the same mutant population and a later developmental stage. This corresponds with previous work by (Panteris and Galatis, 2005) who found that lobing is controlled by microtubule and/or actin filament organisation, where treatment with drugs such as colchicine or oryzalin lead to cellular abnormalities. Several genes have been identified as being important in controlling MC morphology, such as the *SPIKE 1* gene in *Arabidopsis* (Qiu et al., 2002) and the *brick* genes in maize (*Zea mays;* (Frank et al., 2003). Genes relating to mesophyll cell proliferation and expansion are less understood, though provide a promising platform to improve rice photosynthetic productivity (Ren et al., 2019).

### Biological mechanism of stay greenness

Chlorophyll retention under reduced CO_2_ varies greatly both within and between species, with biochemical pathways of photosynthesis providing one explanation as to differences witnessed. Within this study, chlorophyll loss in the C3/C4 intermediate plant *P.miloides* was minimal whilst no chlorophyll loss was witnessed in the C4 *E. glabrescens* plants (**Figure 2**). This aligns with the previous research findings of Menz et al. (1969); Widholm and Ogren (1969) and Cannell et al. (1969) in which plants possessing a Carbon concentrating mechanism (i.e. C4) can survive longer than the less photosynthetically efficient C3 species under sub-ambient CO_2_.

The extent of plant photooxidative damage due to CO_2_ deprivation is assumed to be related to the efficiency in photosynthetic CO_2_ assimilation which is reflected by CO_2_ compensation points. Within *P. milioides*, the mean Γ is ~25 µmol mol^-1^, which is below the treatment concentration of 30 µmol mol^-1^. Therefore, net photosynthesis still occurs, albeit at a reduced rate. However, when the treatment extended for more than 4 days, *P. milioides* plants began to suffer and thus a build-up of photo-oxidative stress may be responsible for the chlorophyll loss witnessed. The biological mechanism responsible for the reduced Γ in *P. milioides* is through a ‘partial functioning C_4_ cycle’ (von Caemmerer, 2000). Within this, a well-developed C_3_ type photosynthesis occurs in mesophyll cells. However, the release of CO_2_ *via* the photorespiratory pathway occurs in bundle sheath cells (BSC) by selective localisation of glycine decarboxylase (Timm et al., 2012) in BSC mitochondria. This released CO_2_ is partially recaptured by BSC chloroplasts, resulting in the reduced Γ. It is predicted that although rice contains a pure C3 pathway, this intermediate type of photosynthesis can be mimicked within rice plants (Furbank et al., 2009).

One of the interesting characteristic features of the selected putative lines, CRM 27 and CRM 29, was an increase in carboxylation rates relative to WT. The exact reason was unclear, however, it is feasible that during the combination of high temperature and reduced CO_2_ may have favoured rice mutants with altered Rubisco properties. CRM 27 also showed significantly lower mitochondrial respiration in the light (Rd*), relative to WT, and had mild changes in Г*. Changes in the Г* are often attributed to differences in the kinetic properties or specificity of Rubisco (Häusler et al., 1999). In transgenic *Arabidopsis* plants where the photorespiratory glycolate pathway had been altered, a reduction in Г* was not associated with a corresponding change in Rd* (Kebeish et al., 2007). This resulted in a reduction in the release of CO_2_ into the intercellular air spaces resulting in a higher A400, and significantly reduced Г. Similarly, Häusler et al. (1999) found that a reduction in Г* is caused by an increased expression level of PEPC enzyme in transgenic potato, which in turn tends to increase Rd* and Rd and suppresses the rate of photorespiration. Other potential candidate genes to alter respiratory pathways could include cyclic electron flow (CEF) or alternative oxidase (*AOX;* Sunil et al., 2019) or improvements to rubisco specificity, flux through the photorespiratory pathway or introducing alternative photorespiratory pathways (for a full review see South et al., 2018).

Mitochondrial respiration rates were measured by different means: ACi curve modelling showed that mean respiration was marginally higher in comparison with WT (**Table 2**) but substantially lower when measured with light response curves (**Table 3**). Moreover, Rd in the three selected CRMs were generally lower than WT (**Supplementary Table 3**). We conclude that we have identified individual mutants with lowered leaf respiration and there are indications that CRM generally have lower Rd (from light response curves), this requires further investigation. Reducing or optimising respiration has had more attention recently as a means of improving productivity whereas previously it has suffered a lack of experimental tractability, however the emergence of high throughput methods are promising (Coast et al., 2019). Evidence now suggests that minimising nocturnal respiration can play a role in optimising carbohydrate consumption especially at higher temperatures (Posch et al., 2019; Xu et al., 2021; Atkin et al., 2023). Despite these findings, Nelson et al. (1975) reported that extended greenness of tall fescue leaves under low CO_2_ is associated with increased respiration, and does not coincide with the changes in Γ. However, this may be due to the less extreme levels of CO_2_ used. Generally, respiration takes place in mitochondria predominantly with the cytochrome pathway (Delgado et al., 1992) although alternative respiratory pathways may also be possible such as the non-phosphorylating mitochondrial electron pathway (Lambers, 1982). The leaf anatomical features also show an important role in recycling respired CO_2_ with plant leaves such as tall fescue and soybean displaying larger and continuous intercellular air spaces. This may enable them to hold larger volumes of respired/photorespired CO_2_ which can potentially be re-utilised in photosynthesis, similar to that seen in *P. milioides*. In contrast, rice leaves typically do not have large intercellular air spaces, although it is possible that mutations impacting leaf cellular anatomy could provide a route to enhancing photosynthetic productivity (Lehmeier et al., 2017; Lundgren et al., 2019).

Manning et al. (1984) illustrated that the prolonged greenness in soybean seedlings under low CO_2_ could be due to the effect of plant hormones or other synthesised compounds, with 2-(4-chlorophenoxy)-2-methyl-propanoic acid (CPMP) reducing leaf chlorophyll loss. CPMP is reported to function in an antagonistic manner to auxin, triggering chloroplast formation and retarding leaf senescence under low CO_2_ conditions as well as increasing leaf chlorophyll content per unit leaf area under non-stressful glasshouse conditions (Wittwer and Murneek, 1946; Burstrom, 1951; Manning et al., 1984). However, whilst CPMP extended greenness under both control and reduced CO_2_ conditions, another compound, 3-butyl-2-hydroxy-4H-pyridol (BHPP) protected against low CO_2_ only (Manning et al., 1984). This suggests that chlorophyll retention under low CO_2_ stress and increased pigmentation under greenhouse conditions are controlled by distinct pathways and thus any improvements to rice photosynthetic performance under low CO_2_ do not necessarily translate to improved performance at ambient levels. Manning et al. (1984) found that the cytokinin, N^6^-benzyladenine (BA) reduced mortality under low CO_2_ but without an extended greenness phenotype in soybean, contrary to previous results in snapbean (Fletcher and Adedipe, 1970). BA is known to maintain RNA and increase protein synthesis. Therefore, a large number of processes can influence chlorophyll retention and/or growth or maintenance under reduced CO_2_.

Alterations in carbohydrate metabolism may contribute to prolonged greenness in rice leaves (Murchie et al., 2009). In plants, the majority of stored starch or sucrose is typically used for growth and metabolic processes during the night, while the remainder is converted to sucrose to fulfil immediate requirements and for export to growing tissues. Thus, continuous carbohydrate starvation under reduced CO_2_ may have led to degradation (yellowing or weakening) of leaf tissue. Alternatively, the possible changes in carbohydrate metabolism pathways may be a result of gene structural changes arising during EMS- or GY- induced mutation.

### Use of high-throughput screening to identify improved metabolic traits

The screening approach utilised here enabled analysis of > 1,000 seedlings per week. This is sufficient to identify required mutational hits, assuming that one reliable mutant could be identified for every 1000 seedling screened (Leung et al., 2001). The identification of 26 putative chlorophyll retention candidates suggests that the rice genome has moderate levels of plasticity to survive under deprived CO_2_ conditions. Correlation analysis indicates that the low CO_2_ screening technique used within this study can be used to identify lines with reduced Γ as well as other potentially beneficial photosynthetic traits (**Figure 3**). As well as A400, Γ also shows significant negative correlations with Amax and CE (r = 0.758, 0.319 and 0.627, respectively). However, Γ is also significantly negatively correlated with gs (r = 0.623), which has implications in terms of water loss.

Screening sensitivity could be further improved by introducing novel technologies. For example, in addition to Γ, C3 and C4 plants can be identified through differences in chlorophyll fluorescence, which can measure changes in photosystem II (PSII) yield (Furbank et al., 2009). Theoretically, when C4 plants are exposed to lower CO_2_ environments (50 to 100 µmol mol^-1^) combined with altered O_2_ levels (from 2% to 21%), O_2_ should not alter PSII yield in C4 plants, whereas C3 plants will exhibit an increase in PSII yield under low O_2_. In practical sense, it is possible to imply these techniques for analysis of individual leaves, however it is necessary to develop new plant screening protocols with these techniques. However, these techniques are emerging such as the screening protocol of McAusland et al. (2019) which utilises chlorophyll fluorescence imaging under controlled gaseous conditions. Following identification of candidate lines from screening, further investigation to be focused on detailed anatomical, plant physiological and DNA sequencing to identify the genetic cause of response.

## Conclusion

Here we present a rapid screening protocol using values from a handheld chlorophyll meter before and after a low CO_2_ treatment to identify candidate mutant rice lines with improved photosynthetic performance. The rice mutants showed mild plasticity to the exposure of sub-optimal CO_2_ levels, and two promising EMS- induced mutant lines were identified with improved performance relative to the WT in terms of respiration and photorespiration. This provides a rapid method to screen for improved photosynthetic performance prior to more detailed anatomical, physiological and genetic analysis.

## Data availability statement

The original contributions presented in the study are included in the article/**Supplementary Material**. Further inquiries can be directed to the corresponding author.

## Author contributions

EM, WQ and AM conceived and designed the experiment. AM performed the experimental work with supervisioin of EM, WQ. AM wrote the initial draft of the paper that was edited by EM and AB. AB performed some of the statistics. KP supervised the cell microscopy. All authors read the manuscript. All authors contributed to the article and approved the submitted version.

## Supplementary material

Supplementary Table 1 List of chlorophyll mutant (CRM lines) for detailed photosynthesis screen.
