# Supplementary material for: Mass screening of rice mutant populations at low CO2 for identification of lowered photorespiration and respiration rates
Source: Front Plant Sci. 2023 Mar 3;14:1125770. doi: 10.3389/fpls.2023.1125770 (PMC10020370; doi:10.3389/fpls.2023.1125770)
Supplement: Supplementary Table 1 — List of chlorophyll mutant (CRM lines) for detailed photosynthesis screen. [file DataSheet_1.zip › Supplementary Data Files.docx]

**Supplementary data file 1**

**Survey of photosynthetic traits in background parental lines and photosynthetically diverse plant species**

The trial was carried out in the glass house facilities at the International Rice Research Institute (IRRI), Manila, the Philippines to analyze the photosynthetic properties of diverse plant species belonging to C_3_, C_3_-C_4_, and C_4_ types to include as control lines in the screen. The parental rice line IR64-21 (C_3_-type), *Panicum miloides* L. (C_3_-C_4_ intermediate type), sorghum (*Sorghum bicolor* (L) Moench*,* C_4_ type), maize (*Zea mayes*) and *Echinochloa glabrescence* L. (C_4_ type) were grown in pots (30cmx30cm x60cm) with the sterilized soil medium. These plants were arranged in complete randomized design with four replicates within the glasshouse. When the plants produced their 6^th^ fully expanded leaves, the photosynthesis analyses were made with an infra-red gas analyser, commercial portable photosynthesis system ( LICOR LI-6400 XT, Nebraska, USA). The assimilation against the internal CO_2_ concentration curves (A/Ci curves) were constructed as per the instructions given by the manufacturer’s manual. Then the curves were analysed with the Microsoft Excel 2007 (Microsoft Corporation, USA). For each of the plant types, the CO_2_ compensation points ( Г ) were calculated as the x- intercept of a linear regression through the five lowest intercellular CO_2_ values on a graph of net CO_2_ assimilation (*A*) versus internal CO_2_ levels (C_i_) (Vogan *et al.,* 2007). The initial slope of *A/Ci* curves were estimated for slopes, as maximum carboxylation efficiency (CE) and overall respiration rate (Ro) estimated by interpolation of liner regression to the Y-axis. Then maximum photosynthetic rate the leaf can attain at saturated light with the provision of 400 µmol mol^-1^ CO_2_ levels is referred as ambient level (A_400_) were also estimated from these A/Ci curves.

A comparison of gas exchange parameters for background parental lines were made. Here, we tested the response of three-types of pathways the C_3_, C_4_ and C_3_-C_4_ intermediates. Typically, the IR64-21 is the parental rice line for rice deletion mutants follows C_3_-type, *Panicum miloides* is a C_3_-C_4_ intermediate type, whereas sorghum, (*Sorghum bicolor),* maize (*Zea maiz*) and *E.* *glabrescens* strictly follows of C_4_ type. The reason for testing *E. glabrescens* photosynthesis parameters in the present study was that *E. glabrescens* requires same ecological growth conditions as rice, such as high water-logged conditions coupled with increased growth temperatures ( above 28 ^o^C) and they grow as an invasive C_4_ weed in rice fields.

**Table 2.** Photosynthetic properties in the 5^th^ leaf of background plant species. The CO_2_ compensation point (Г) carboxylation efficiencies (CE) and estimated overall respiratory rates (Ro) in IR64 parental lines, *Panicum miloides* , maize, sorghum and *E. glabrescens* are showed. SEM- standard error of mean. Asterisks represent the significance levels when comparing with IR64 plants. * P<0.05, ** P<0.01, n = number of plants tested in the experiment.


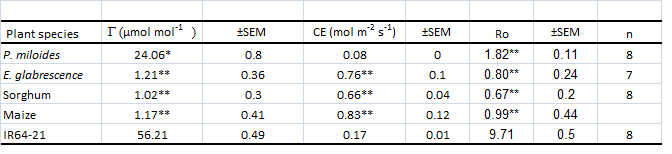


As shown in Table 2, the rice plants possessed significantly increased Г than the C_4_ and C_3_-C_4_ intermediates with an average Г of 56.2 ±0.48 µmol mol^-1^. In contrast for *P. miloides* (C_3_-C_4_ intermediate) displayed Г value was 24 µmol mol^-1^, while in typical C_4_ plants such as maize and sorghum showed Г below 5 µmol mol^-1^, suggesting that the CO_2_ compensation point can be used as one of the powerful tool to discriminate plant species. As such, this trait can be used as a criterion for screening of photosynthetically efficient plants.

Distinguishable variations were also be seen for carboxylation efficiencies among the tested lines. The carboxylation efficiencies of Rubisco enzyme were measured from the slope of A/Ci response curves. The C_4_ plants showed significantly increased CE than rice plants. However, in P. *miloides*, displayed insignificant differences in CE when compared to the rice plants, although they possessed lower CO_2_ compensation point. The overall estimated respiratory rates (dark + light) of plants were compared. C_4_ plants had significantly lower Ro than the wild type rice plants. Similarly, *P.miloides* also possessed lower rates of Ro than the wild type rice counterparts.

**Supplementary data file 2**

**Components of a CO_2_ chamber**

The re-modified screening chambers (1mx1mx1m) at IRRI were designed by John Sheehy *et al*. (IRRI, 2008) after series of discussion with Menz *et al* (1969) for screening of wild rice species at lower CO_2_ levels (IRRI, unpublished data). The chamber consists of three-primary structures, they are Dome, Cover-lid and well-base (Figure 1). The dome serves as the structure to hold the high-density discharge lamps which are used in day times on cloudy days and throughout the night. The dome also holds a water-pan in order to absorb the heat of the lamps. The cover lid is made of ½” transparent acrylic and serves as air tight unit in the chamber. The well-base is used to keep seedlings and reserves for supply of nutrient solution to the plants (Yoshida 1981).

**
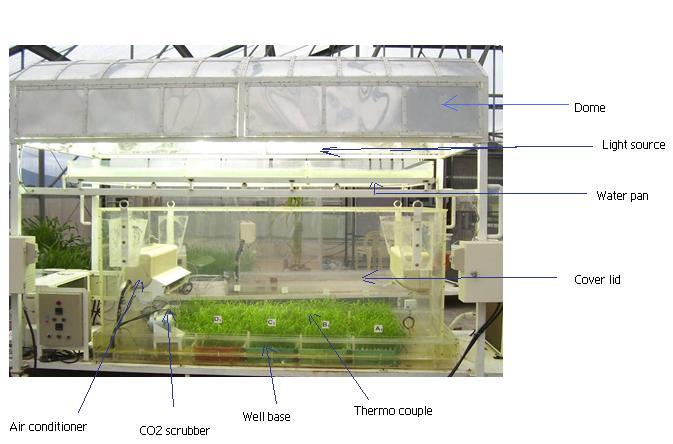
**

**Figure 1.** Structure of High-throughput CO_2_ screening chamber. This consists of three major components viz; Dome, cover lid and well base of chamber. Designed and re-modified by John Sheehy *et al.* (2008).

Mechanisms of CO_2_ control in the chamber

The CO_2_ levels in the chamber were monitored with 4-major sub units. Viz: WMA-4 analyser a non-dispersive infrared gas analyzer, CO_2_ control board, CO_2_ scrubbing unit and the solenoid switch. WMA-4 CO_2_ analyser (PP Systems, Hitchin, UK) monitors the CO_2_ levels (ppm) within the chamber and provides electronic signals to the CO_2_ controller unit, which is fixed in main console. When the CO_2_ level rises above the set value in the chamber, the control board sends signals to the CO_2_ scrubber (a suction pump which passes through the air through soda lime). When the CO_2_ levels goes below the set values in chamber, solenoid valve receives signals from CO_2_ control board and the solenoid switch allows fresh air to flow inside the chamber. Relative humidity (RH%) and temperatures were monitored with computerised units of the chamber. Generally the RH % was maintained 60% to 65% and the temperature at 28^o^ C, the temperature probes positioned inside the chamber (cover-lid) which were connected to an air-conditioners or air chillier systems.  A constant air flow rate (0.4L/minute) which was maintained with a flow metre through the 4 days of treatment. In terms of CO_2_ treatment regimes, the 1^st^ day maintained at 60 ppm and the remainder three-days maintained at 30ppm levels.


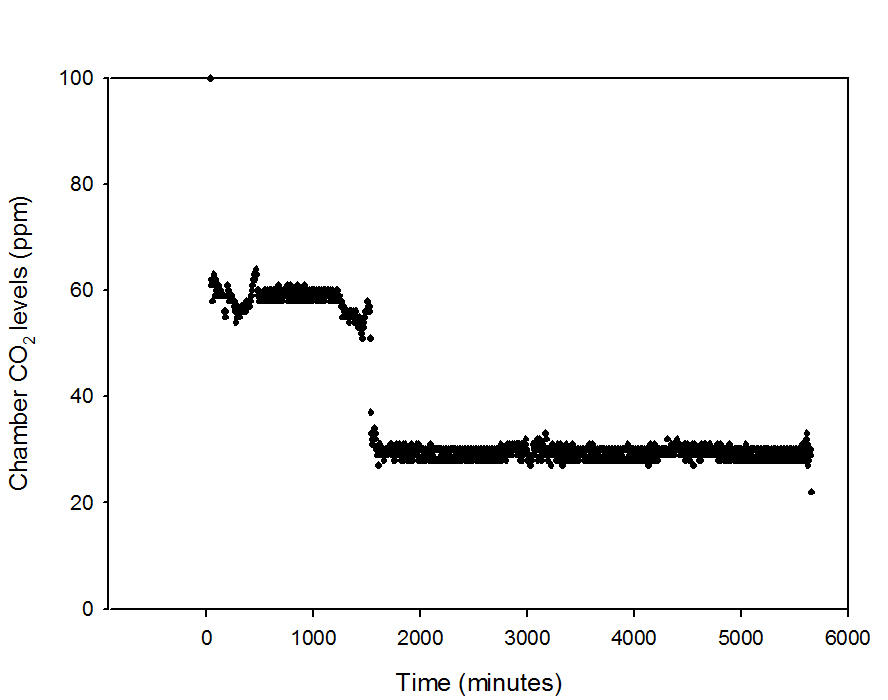


**Figure 2.** CO_2_ levels in chambers over the 4-days of treatment. It maintained around 60ppm on the 1^st^ day, then 30ppm for the next 3-days. The CO2 levels were monitored using WMA-CO_2_ analyzers.
