# Supplementary material for: Mass screening of rice mutant populations at low CO2 for identification of lowered photorespiration and respiration rates
Source: Front Plant Sci. 2023 Mar 3;14:1125770. doi: 10.3389/fpls.2023.1125770 (PMC10020370; doi:10.3389/fpls.2023.1125770)
Supplement: Supplementary Table 1 — List of chlorophyll mutant (CRM lines) for detailed photosynthesis screen. [file DataSheet_1.zip › Supplementary Table 1.docx]

**Supplementary Table 1.** List of chlorophyll mutant (CRM lines) for detailed photosynthesis screen

| **IR64 Family** | **CRM line** | **Mutagen** |
| --- | --- | --- |
| IR64 | WT | Control plant |
| IR 64 E 26887-1-B-3 | CRM 24 | Ethyl methonosulfonate |
| IR 64 E 26958-1-B-1 | CRM 25 | Ethyl methonosulfonate |
| IR 64 E 26958-1-B-2 | CRM 26 | Ethyl methonosulfonate |
| IR 64 E 27054-1-B-7 | CRM 27 | Ethyl methonosulfonate |
| IR 64 E 27142-1-B-4 | CRM 29 | Ethyl methonosulfonate |
| IR 64 E 27472-1-B-2 | CRM 31 | Ethyl methonosulfonate |
| IR 64 E 27472-1-B-3 | CRM 32 | Ethyl methonosulfonate |
| IR 64 E 27472-1-B-4 | CRM 33 | Ethyl methonosulfonate |
| IR 64 G 16692-1-B-1 | CRM 34 | Gamma irradiance |
| IR 64 G 16709-1-B-3 | CRM 35 | Gamma irradiance |
| IR 64 G 16722-1-B-1 | CRM 36 | Gamma irradiance |
| IR 64 G 16767-1-B-8 | CRM 37 | Gamma irradiance |
| IR 64 G 16965-1-B-1 | CRM 38 | Gamma irradiance |
| IR 64 G 17500-1-B-3 | CRM 39 | Gamma irradiance |
| IR 64 G 17500-1-B-5 | CRM 40 | Gamma irradiance |
| IR 64 G 17532-1-B-10 | CRM 41 | Gamma irradiance |
| IR 64 G 17545-1-B-1 | CRM 42 | Gamma irradiance |
| IR 64 G 17565-1-B-1 | CRM 43 | Gamma irradiance |
| IR 64 G 17565-1-B-6 | CRM 44 | Gamma irradiance |
| IR 64 G 18100-1-B-1 | CRM 55 | Gamma irradiance |
| IR 64 G 18112-1-B-6 | CRM 56 | Gamma irradiance |
| IR 64 G 19636-1-B-4 | CRM 58 | Gamma irradiance |
| IR 64 G 20435-1-B-1 | CRM 64 | Gamma irradiance |
| IR 64 G 20435-1-B-4 | CRM 65 | Gamma irradiance |
| IR 64 G 21087-1-B-5 | CRM 67 | Gamma irradiance |
| IR 64 G 21139-1-B-1 | CRM 68 | Gamma irradiance |
