# Supplementary material for: Mass screening of rice mutant populations at low CO2 for identification of lowered photorespiration and respiration rates
Source: Front Plant Sci. 2023 Mar 3;14:1125770. doi: 10.3389/fpls.2023.1125770 (PMC10020370; doi:10.3389/fpls.2023.1125770)
Supplement: Supplementary Table 1 — List of chlorophyll mutant (CRM lines) for detailed photosynthesis screen. [file DataSheet_1.zip › Supplementary Table 2.docx]

**Supplementary Table 2.**

Photosynthetic traits of chlorophyll retention mutant (CRM) rice lines relative to the parental wild type (WT) IR64 from spot measurements and ACi curves. Means and standard error of the means are shown where n≥3 for A400 (μmol m^−2^ s^−1^): Photosynthetic rate at 400 ppm CO_2_ concentration; Г (μmol mol^−1^): CO_2_ compensation point; CE (mol m^−2^ s^−1^): carboxylation efficiency; Ci (μmol mol^−1^): intercellular CO_2_ concentration; *gs* (mmol m^−2^ s^−1^): Stomatal conductance. * and ** indicates significant differences between lines according to ANOVA at P < 0.01 and P < 0.001, respectively (p<0.05). Letters indicate differences according to post-hoc Bonferroni test.

| **Genotype** | **A400**** | **Ci*** | **gs**** | **Г**** | **CE**** | **Ro**** |
| --- | --- | --- | --- | --- | --- | --- |
| **WT** | 22.15±0.78*^bcd^* | 314.60±7.07 | 0.59±0.03*^abcdefg^* | 58.76±1.67*^a^* | 0.11±0.01*^abc^* | 6.45±0.25*^abcd^* |
| **CRM 24** | 20.96±0.72*^abcd^* | 326.07±1.72 | 0.60±0.03*^abcdefgh^* | 65.58±1.58*^ab^* | 0.10±0.01*^abc^* | 6.35±0.36*^abcd^* |
| **CRM 25** | 19.31±0.77*^abc^* | 322.39±6.85 | 0.55±0.06*^abcde^* | 66.22±4.51*^ab^* | 0.09±0.01*^abc^* | 6.20±0.44*^abcd^* |
| **CRM 26** | 25.93±0.94*^bcd^* | 337.30±2.09 | 0.88±0.05*^befgh^* | 55.19±2.34*^a^* | 0.15±0.01*^c^* | 8.41±0.64*^bcd^* |
| **CRM 27** | 25.40±1.79*^bcd^* | 305.55±7.12 | 0.58±0.05*^abcdef^* | 57.25±3.04*^a^* | 0.15±0.01*^c^* | 8.57±0.27*^bd^* |
| **CRM 29** | 24.65±0.62*^bcd^* | 339.90±3.40 | 0.95±0.05*^fh^* | 49.02±0.31*^a^* | 0.12±0.01*^abc^* | 5.75±0.30*^abcd^* |
| **CRM 31** | 22.58±1.49*^abcd^* | 313.06±4.67 | 0.50±0.03*^abc^* | 60.32±3.11*^ab^* | 0.12±0.01*^abc^* | 7.54±0.74*^abcd^* |
| **CRM 32** | 20.95±0.83*^abcd^* | 320.43±4.37 | 0.53±0.03*^abcd^* | 64.97±3.65*^ab^* | 0.10±0.00*^abc^* | 6.26±0.16*^abcd^* |
| **CRM 33** | 22.19±0.60*^abcd^* | 319.88±3.28 | 0.57±0.03*^abcde^* | 59.87±1.08*^ab^* | 0.11±0.01*^abc^* | 6.53±0.37*^abcd^* |
| **CRM 34** | 19.08±1.37*^ab^* | 313.78±7.77 | 0.47±0.08*^a^* | 61.68±0.77*^ab^* | 0.09±0.01*^abc^* | 5.55±0.53*^abc^* |
| **CRM 35** | 13.90±4.30*^a^* | 291.96±24.31 | 0.38±0.07*^a^* | 79.83±9.74*^b^* | 0.08±0.02*^a^* | 5.56±1.02*^abc^* |
| **CRM 36** | 21.44±1.21*^abcd^* | 322.50±4.10 | 0.58±0.06*^abcdef^* | 61.75±1.46*^ab^* | 0.13±0.01*^abc^* | 6.78±0.80*^abcd^* |
| **CRM 37** | 22.78±1.02*^abcd^* | 324.87±5.24 | 0.70±0.04*^abcdefgh^* | 53.27±1.59*^a^* | 0.12±0.01*^abc^* | 6.24±0.74*^abcd^* |
| **CRM 38** | 25.19±1.13*^bcd^* | 307.48±8.36 | 0.62±0.04*^abcdefgh^* | 63.99±6.77*^ab^* | 0.13±0.01*^abc^* | 8.40±0.37*^bcd^* |
| **CRM 39** | 24.39±1.66*^bcd^* | 323.10±2.72 | 0.64±0.03*^ab^* | 61.14±2.83*^ab^* | 0.14±0.01*^bc^* | 8.50±0.59*^bcd^* |
| **CRM 40** | 23.66±1.17*^bcd^* | 318.78±8.50 | 0.66±0.13*^abcdefgh^* | 55.24±2.59*^a^* | 0.12±0.01*^abc^* | 6.49±0.45*^abcd^* |
| **CRM 41** | 24.19±0.84*^bcd^* | 312.17±1.93 | 0.56±0.03*^abcde^* | 55.90±1.64*^a^* | 0.13±0.01*^abc^* | 7.21±0.56*^abcd^* |
| **CRM 42** | 21.95±2.25*^abcd^* | 296.23±13.40 | 0.48±0.12*^a^* | 62.32±4.94*^ab^* | 0.11±0.01*^abc^* | 6.78±0.64*^abcd^* |
| **CRM 44** | 21.50±1.76*^abcd^* | 305.91±4.36 | 0.44±0.04*^a^* | 60.75±2.78*^ab^* | 0.11±0.01*^abc^* | 6.70±0.59*^abcd^* |
| **CRM 54** | 18.85±4.10*^ab^* | 319.09±11.84 | 0.49±0.14*^ab^* | 69.87±6.56*^ab^* | 0.08±0.03*^ab^* | 5.35±1.39*^ab^* |
| **CRM 55** | 21.01±1.36*^abcd^* | 337.88±2.70 | 0.72±0.03*^abcdefgh^* | 66.71±6.31*^ab^* | 0.10±0.00*^abc^* | 6.67±0.63*^abcd^* |
| **CRM 56** | 23.29±0.56*^bcd^* | 326.14±1.54 | 0.64±0.03*^abcdefgh^* | 62.50±1.96*^ab^* | 0.11±0.00*^abc^* | 7.01±0.26*^abcd^* |
| **CRM 58** | 23.69±0.99*^bcd^* | 325.01±4.31 | 0.66±0.05*^abcdefgh^* | 60.95±1.46*^ab^* | 0.12±0.01*^abc^* | 7.29±0.34*^abcd^* |
| **CRM 64** | 20.66±2.63*^abcd^* | 309.11±13.44 | 0.48±0.11*^ab^* | 63.06±8.95*^ab^* | 0.08±0.03*^ab^* | 4.73±1.44*^a^* |
| **CRM 65** | 28.18±1.89*^bd^* | 331.61±3.97 | 0.87±0.04*^bdefgh^* | 49.19±0.77*^a^* | 0.15±0.01*^c^* | 7.40±0.61*^abcd^* |
| **CRM 67** | 23.90±1.61*^bcd^* | 324.61±6.99 | 0.70±0.04*^abcdefgh^* | 52.20±0.99*^a^* | 0.13±0.00*^abc^* | 6.71±0.10*^abcd^* |
| **CRM 68** | 22.27±1.57*^abcd^* | 314.15±5.47 | 0.55±0.07*^abcde^* | 59.17±4.63*^ab^* | 0.10±0.01*^abc^* | 5.82±0.23*^abcd^* |
| **Mean CRM** | **22.38±0.5** | **318.46±2.32** | **0.61±0.03** | **60.69±1.31** | **0.11±0.004** | **6.72±0.20** |
