# Supplementary material for: Mass screening of rice mutant populations at low CO2 for identification of lowered photorespiration and respiration rates
Source: Front Plant Sci. 2023 Mar 3;14:1125770. doi: 10.3389/fpls.2023.1125770 (PMC10020370; doi:10.3389/fpls.2023.1125770)
Supplement: Supplementary Table 1 — List of chlorophyll mutant (CRM lines) for detailed photosynthesis screen. [file DataSheet_1.zip › Table 3.pdf]

Supplementary Table 3

Photosynthetic traits of chlorophyll retention mutant (CRM) rice lines relative to the parental wild type (WT) IR64-21 from light response curves (LRCs).

Genotype	A_{max}**	QE**	LCP*	LSP*	Rd
**WT**	38.04±1.04^{bc}	0.06±0.00^{b}	42.68±2.71^{abcd}	688.17±28.53^{abcd}	2.58±0.21
**CRM 27**	36.63±0.78^{bc}	0.05±0.00^{ab}	34.50±2.23^{abc}	834.43±43.53^{abcde}	1.63±0.18
**CRM 29**	42.60±0.15^{bc}	0.06±0.00^{b}	34.27±4.94^{ab}	702.00±15.04^{abcde}	2.20±0.35
**CRM 31**	39.90±3.42^{bc}	0.06±0.01^{b}	34.33±2.02^{ab}	730.50±110.63^{abcde}	2.08±0.34
**CRM 32**	36.36±2.83^{bc}	0.06±0.01^{b}	37.26±7.23^{abcd}	654.40±43.49^{ab}	2.08±0.22
**CRM 33**	34.86±1.17^{bc}	0.06±0.00^{b}	32.98±1.82^{ab}	642.20±29.36^{ab}	1.88±0.07
**CRM 34**	34.30±2.27^{bc}	0.06±0.01^{ab}	27.33±3.99^{a}	689.50±84.67^{abcde}	1.59±0.39
**CRM 35**	21.00±2.54^{a}	0.02±0.00^{a}	62.35±15.58^{bd}	944.20±19.67^{abcde}	1.67±0.33
**CRM 36**	35.83±2.85^{bc}	0.06±0.01^{b}	32.15±2.07^{ab}	681.00±35.58^{abc}	1.77±0.13
**CRM 37**	32.90±6.16^{abc}	0.05±0.01^{ab}	49.20±2.77^{abcd}	773.67±98.00^{abcde}	2.34±0.61
**CRM 40**	29.93±1.88^{ab}	0.04±0.00^{ab}	46.10±9.04^{abcd}	828.75±38.58^{abcde}	1.70±0.24
**CRM 43**	48.57±6.40^{c}	0.05±0.01^{ab}	31.47±4.62^{ab}	1025.67±184.79^{ace}	1.71±0.58
**CRM 68**	31.10±4.20^{ab}	0.04±0.01^{ab}	52.53±2.20^{abcd}	926.33±144.18^{abcde}	2.03±0.58
**Mean CRM**	**35.33±1.97**	**0.05±0**	**39.55±3.04**	**785.67±36.38**	**1.89±0.07**

Means and standard error of the means are shown where n≥3 for A_{max} (µmol m^{-2} s^{-1}): maximum assimilation rate; QE (mol mol^{-1}): quantum use efficiency; LCP (µmol m^{-2} s^{-1}): light compensation point; LSP (µmol m^{-2} s^{-1}): light saturation point and; Rd (µmol m^{-2} s^{-1}): dark respiration. * and ** indicates significant differences between lines according to ANOVA at $P < 0.01$ and $P < 0.001$, respectively ($p<0.05$). Letters indicating differences according to post-hoc Bonferroni test.
